# Letter from Dr. J. W. Daniel

**Published:** 1896-10

**Authors:** J. W. Daniel

**Affiliations:** Houston, Texas


**A New and Useful Treatment for Gonorrhœa.—Letter from Dr. J. W. Daniel.**

HOUSTON, TEXAS, June 23, 1896.

*Dr. F. E. Daniel, Editor Texas Medical Journal, Austin, Texas:*

* * * I have been having unusually good success in the treatment of gonorrhœa lately with the new substance argentum-casein (argonin), recently introduced by Dr. A. Jadssohn, notice of which has been given in recent medical literature, and

I feel so elated over my success that I am itching all over to tell about it. Without going into details, I will say that so far I have treated eight cases of the disease in the acute stage and six in the more advanced, or what is usually denominated the stationary stage of the disease. Nitrate of silver has always been a favorite remedy with me in gonorrhœa, but I had almost abandoned the use of the drug on account of its irritating properties, only using it in those posterior urethral complications which we meet with in old chronic cases. In these conditions the nitrate is the ideal remedy. A preparation of the nitrate of silver having all the curative properties of that drug, stripped of its irritating nature, caught my fancy at once, and I immediately began the use of it. An injection containing 12 grains of argonin to the ounce of water was ordered, and used at once without any reference to the stage of the disease. In all of those cases where the inflammatory conditions were most painfully prominent the drug acted like a charm, the inflammation subsided, the scalding in passing the water was lessened, and the discharge changed, both in color and consistency, ceasing entirely on the fifth and sixth days after beginning the use of the remedy. The injection was used six or seven times during the twenty-four hours. The patient was directed to flush the urethra with water after micturition, so as to clear the canal of urine, and then inject the medicine. I noticed the improvement in my acute cases at once. The discharge was examined from time to time during the first six days, and the gonococci were found to be growing less at each examination, until they had entirely disappeared in four cases on the fourth day from beginning of treatment, in two on the fifth, one on the sixth, and one on the ninth. In this last case, however, the gonococci reappeared on the fifteenth day. This case was an old stager, he having had gonorrhœa several times before. I suspected a narrowing of the canal from previous disease, which was readily defined on using the sound. This case is still under treatment. The other seven acute cases were directed to use the remedy in half strength for five or six days as a precautionary measure when they were discharged. The average duration of treatment in the seven cases was fourteen days, although the discharge had ceased on the sixth day entirely. In the more advanced stages of the disease, where it had existed for several weeks, the results have not been so decided; however, there has been considerable improvement. These are usually old hands at the bellows, parties who have had the disease several times before, and whose condition is complicated more or less by strictures and granular thickening of the urethral membrane, which render their cases obstinate and difficult to treat. These cases are still under treatment. The duration of their treatment so far will cover a period of from eleven to twenty-three days.

So many new remedies, "dead shots," "three day cures," for gonorrhœa have come under our observation in the past that we naturally approach these new candidates for our favors with a great deal of caution, and I am even now, with the gratifying results that have followed the use of argonin, a little reluctant to urge its claims until I have given it a more extended trial. Later on we will have something more to say about argonin.

Yours truly,

J. W. DANIEL, M. D.

---

### Recent Meeting of the American Ophthalmological Society at New London, Conn.

DALLAS, TEXAS, September 25, 1896.

*Editor Texas Medical Journal:*

